# Antibiotic prophylaxis for infections in patients with acute stroke: a systematic review and meta-analysis of randomized controlled trials

**DOI:** 10.18632/oncotarget.19039

**Published:** 2017-07-06

**Authors:** Yan-Guo Xi, Xu Tian, Wei-Qing Chen, Sai Zhang, Shan Zhang, Wei-Dan Ren, Qi-Jun Pang, Guo-Tao Yang, Zhi-Ming Yang

**Affiliations:** ^1^ Department of Neurosurgery, Cang Zhou Central Hospital, Hebei 061001, China; ^2^ Department of Gastroenterology, Chongqing Cancer Institute and Hospital and Cancer Center, Chongqing 400030, China; ^3^ Department of Neurosurgery, Logistic University Affiliated Hospital, Logistic University of Chinese People's Armed Police Force, Tianjin 300162, China

**Keywords:** acute stroke, antibiotic prophylaxis, systematic review, meta-analysis

## Abstract

**Objective:**

Infections are frequent after stroke and lead to increased mortality and neurological disability. Antibiotic prophylaxis has potential of decreasing the risk of infections and mortality and improving poor functional outcome. Several studies evaluated antibiotic prophylaxis for infections in acute stroke patients have generated conflicting results. The systematic review of randomized clinical trials (RCTs) aimed at comprehensively assessing the evidence of antibiotic prophylaxis for the treatment of acute stroke patients.

**Materials and Methods:**

PubMed, EMBASE, the Cochrane library and the reference lists of eligible articles were searched to identify all potential studies. We included the studies that investigated the efficacy and safety of antibiotic prophylaxis for the treatment of acute stroke patients. The primary outcome included mortality and infection rate. The secondary outcomes included poor functional outcome and adverse events.

**Results:**

Seven trials randomizing 4,261 patients were included. Pooled analyses showed that antibiotic prophylaxis did not improve the mortality (risk ratio (RR) = 1.03, 95% confidence interval (CI) 0.84 to 1.26, *p* = 0.78, *I*^2^ = 25%) and poor functional outcome (RR = 0.93, 95% CI 0.80 to 1.08, *p* = 0.32, *I*^2^ = 80%), but reduced the incidence of infection (RR = 0.67, 95% CI 0.53 to 0.84, *p* = 0.0007, *I*^2^ = 49%). No major side effects were reported. Sensitivity analyses confirmed the results of infection rate and poor functional outcome.

**Conclusions:**

Antibiotic prophylaxis can be used to treat the infectious events of acute stroke patients although it has no potential of decreased mortality and improved functional outcome.

## INTRODUCTION

Stroke is a major contributor to cardiocerebral vascular diseases-related disability and death worldwide, especially in low- and middle-income countries [1, 2]. Stroke not only impairs many vital neurological functions, but causes severe complications such as infections [3]. Previous definitions used for diagnosing infection were substantially different, and hence it was defined predominately based on the modified Centers for Disease Control and Prevention criteria [4]. About 5–65% of acute stroke patients are at risk of infection [5]. A meta-analysis including 137,817 patients reported an overall infection rate of 30%, as for pneumonia and urinary tract infection (UTI), the corresponding rate was 10% [5]. In intensive care units (ICU), the infection rate was up to 45% [5]. Of patients with post-stroke infection, 48% died compared to 18% patients without infection [5]. Several studies well demonstrated that post-stroke infection was associated with increased mortality and poor functional outcome [6, 7]. Thus, it is extremely important to effectively and successfully manage the infection following the stroke.

Published evidences demonstrated that antibiotic prophylaxis therapy may decrease the risk of infection [8] and mortality [9] and improve the functional outcome. Previous experimental studies [10, 11] demonstrated that antibiotic prophylaxis reduced post-stroke infection and also improved other clinical outcomes. Moreover, several animal studies [12–14] also suggested that some antibiotics have a potential neuroprotective effect. Of these antibiotics, Minocycline and ceftriaxone have been shown to improve neurological performance and survival through inducing glutamate transporter expression, stimulating neurotrophins or suppressing the release of inflammatory cytokines. However, the relevant clinical trials of investigated the efficacy of antibiotic prophylaxis in treating acute stroke patients generated conflicting findings [9, 15–20]. The PANTHERIS trial [16], a randomized, double-blind, and placebo-controlled trial, recruited 79 patients who suffered acute ischemic stroke with a National Institute of Health Stroke Scale (NIHSS) score > 11. Administration of moxifloxacin was intravenously initiated within 36 hours after stroke onset, with 400 mg daily for 5 days. Moxifloxacin reduced the infection rate from 32.5% to 15.4% but did not significantly improve neurological outcome and survival. Lampl [18] performed another randomized controlled trial (RCT) in Israel, in which they recruited 141 patients with NHISS > 5 under 5-day taking Minocycline treatment at a dosage of 200 mg. The result illustrated that, compared with placebo, patients assigned to minocycline obtained superior outcome on the followed 7th day and 30th day. Chamorro [15] also designed a RCT in Spain, which recruited 136 patients with a NIHSS ≥ 5 for intravenous levofloxacin treatment of 3-day duration. Controversial results were reported that prophylactic administration of levofloxacin was not better than optimal care. It is must be noted that the current guidelines did not recommend antibiotic prophylaxis to treat acute stroke patients [21]. More importantly, a recent large-scale study suggested [17] that antibiotic prophylaxis did not reduce incidence of post-stroke pneumonia in stroke patients with dysphagia. Two prospective and multicenter trials also concluded that antibiotic prophylaxis did not improve functional outcome at 3 months in acute stroke patients [17, 19]. As a result, it remains controversial whether antibiotic prophylaxis has the potential of reducing rate of infections, mortality and disability in stroke patients. And thus, we designed this systematic review to comprehensively assess the effects of antibiotic prophylaxis on post- stroke infections and functional outcome in acute stroke patients.

## RESULTS

### Description of the studies

**Figure 1:**
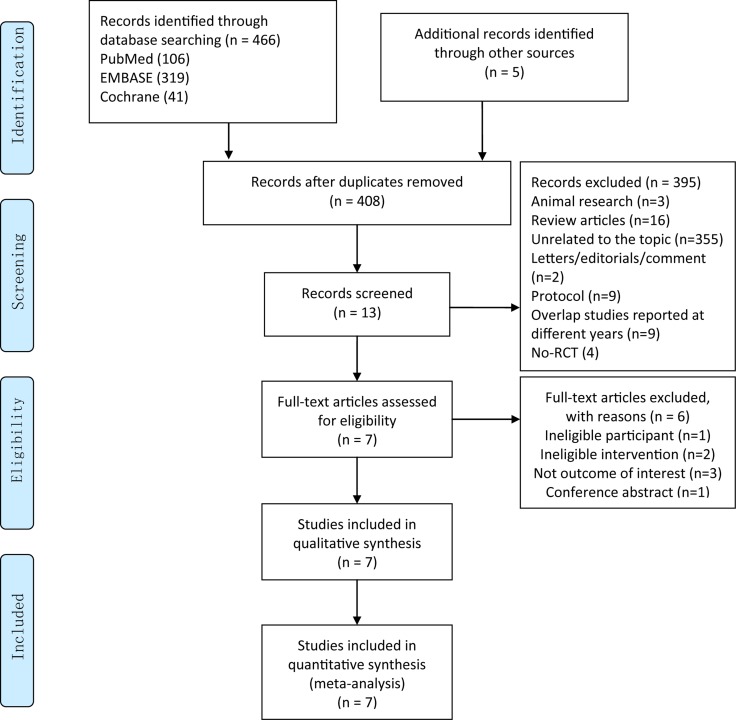
Flow chart of retrieval and selection of literatures RCT = randomized controlled trials.

We initially captured 471 records and 13 articles were included to evaluate the eligibility based on full-text. Six studies were excluded because of ineligible participant, ineligible intervention and lack of eligible outcomes of interest and 7 RCTs were included into qualitative synthesis eventually. The Figure 1 showed the process of searching and screening of studies.

Characteristics of included studies are documented in Table 1. These studies [9, 15–20] enrolling 4,261 stroke patients (2131 in antibiotics groups versus 2130 in control groups). The sample size of individual study varied from 60 to 2538. In the control groups, 228 patients [15, 16, 18, 20] were randomized to receive placebo and 1902 patients [9, 17, 19] have no additional treatment. The basic median stroke severity scores were based on the National Institute of Health Stroke Scale (NIHSS) in six studies [9, 15–19]. These scores ranged from 7.6 to 15 in control groups versus 7.5 to 17 in antibiotics groups. The Canadian Neurological Scale (CNS) was used to assess stroke severity in one study [20] (median score of 4.1 in control groups versus 4.5 in antibiotic groups). Three studies [15, 17, 19] enrolled ischemic and hemorrhagic stroke patients, whereas the remaining four studies [9, 16, 18, 20] only recruited ischemic stroke patients. Intervention regimes differed in these seven studies: fluoroquinolones (levofloxacin [15], moxifloxacin [16]) were used in two studies, ceftriaxone [19] in one study, penicillin [20] in one study, minocycline [18] in one study, amoxicillin or co-amoxiclav, together with clarithromycin [17] in one study and a combination of β-lactam antibiotic with β-lactamase inhibitor [9] in one study. Route of administration included intravenous [9, 15–17, 19], intramuscular [20] and oral [18]. Duration of preventive antibiotic therapy ranged from 3 to 7 days and was not noted in one study [20].

**Table 1:** Characteristics of the included studies

Author (year)	Study design	Inclusion criteria	Exclusion Criteria	Sample	Treatment arms (No)	Intervention	Outcomes of interest
Chamorro 2005^15^	RCT double-blind	Ischemic stroke < 12 hours; Age ≥ 18 years, (NIHSS) ≥ 5,	Infection < 3 mo; T > 37. 7°C; allergy to fluoroquinolones; epilepsy; seizures; serum creatinine > 2.5 mg/dL, antibiotics use; immunosuppressants therapy < 3 mo	136	Antibiotics (67) vs placebo (69)	Levofloxacin 500 mg/d IV for 3d, started within 24 h of stroke onset	7-day infection, 3-month neurological outcome and mortality
Harms 2008^16^	RCT double-blind	Ischemic stroke (9–36 hours); Age ≥ 18 years, NIHSS > 11	infections; Hemorrhagic stroke; antibiotics therapy < 24 h; contraindications against moxifloxacin; immunosuppressant treatment	79	Antibiotics (39) vs placebo (40)	Moxifloxacin 400 mg/d IV for 5 d, started within 36 h of stroke onset	11-day infection, 6-month neurological outcome and mortality
Kalra 2015^17^	RCT open-label	Stroke < 48 hours; Age ≥ 18 years, with dysphagia	Infections; allergic to antibiotics; preexisting dysphagia; pyrexia; pregnancy; imminent death	1217	Antibiotics (615) vs control (602)	Amoxicillin or co-amoxiclav, together with clarithromycin IV for 7 d, started within 48 h of stroke onset	14-day infection, 3-month neurological outcome and mortality
De Falco 1998^20^	RCT open-label	Ischemic stroke < 12 hours; All ages	NA	80	Antibiotics (38) vs control (42)	Penicillin intramuscularly, started within 12 h of stroke onset	In-hospital infection and mortality
Lampl 2007^18^	RCT open-label	Ischemic stroke (6–24 hours); Age ≥ 18 years, NIHSS > 5	Hemorrhagic stroke; other disease; pre-existing neurologicdisability; tetracycline allergic; renal failure; pre- existing infectious disease; swallowing difficulties	151	Antibiotics (74) vs placebo (77)	Minocycline 200mg/d orally for 5 d, started within 6–24 h of stroke onset	3-month neurological outcome and mortality
Schwarz 2008^9^	RCT open-label	Ischemic stroke < 24 hours; Age ≥ 18 years, mRS > 3	Infections; hemorrhagic stroke; renal insufficiency; penicillin or sulbactam allergic; immunosuppressant treatment; pregnancy	60	Antibiotics (30) vs control (30)	Mezlocillin 6g/d plus sulbactam 1g/d IVfor 4d, started within 24 h of stroke onset	10-day infection, 3-month neurological outcome and mortality
Westendorp 2015^19^	RCT open-label	Stroke < 24 hours; Age ≥ 18 years, NIHSS ≥ 1	Infections; antibiotics therapy < 24 h; pregnancy; penicillin or cephalosporins allergic; subarachnoid hemorrhage;	2538	Antibiotics (1268) vs control (1270)	Ceftriaxone 2 g/d IV for 4 d, started within 24 h of stroke onset	In-hospital infection, 3-month neurological outcome and mortality

RCTs = randomized controlled trials; NIHSS = National Institute of Health Stroke Scale; mRS = modified Rankin Score; vs = versus; mo = month; t = temperature; NA = not available.

### Quality assessment

**Figure 2:**
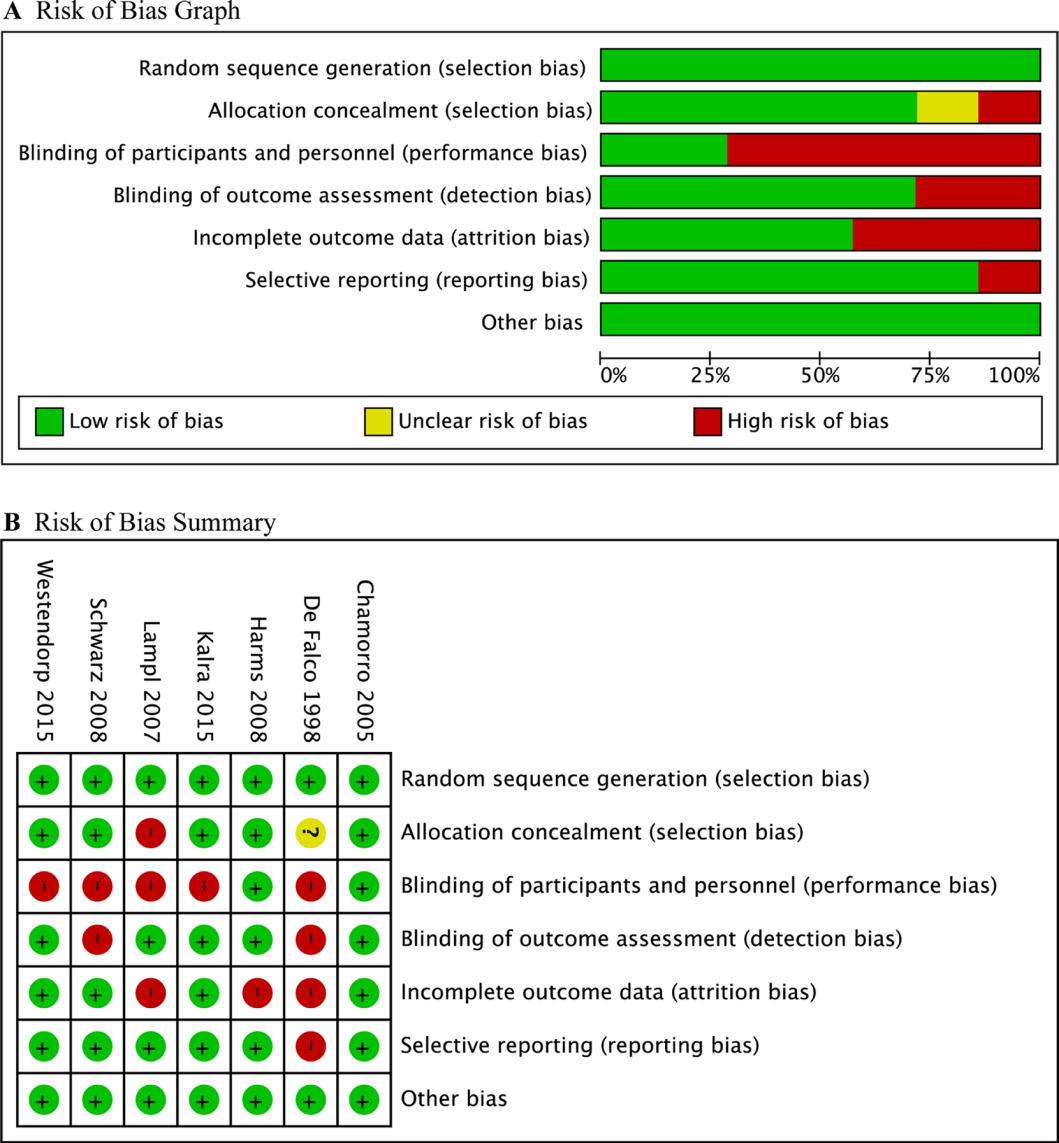
Risk of bias (**A**). Risk of bias graph, (**B**) Risk of bias summary. Green, yellow, and red color indicated low, unclear, and high risk of bias respectively.

The assessment of the risk of bias for individual studies is delineated in Figure 2. All studies generated random sequence appropriately. Allocation concealment was not referred in one study [20]. One study used the 8th number of the participant's identity card (ID) for randomization, which could be concluded that treatment allocation was not concealed because physicians could know patient's ID numbers and get a certain treatment. Due to open-label design, 4 [9, 17–19] out of 7 studies have performance bias. Of these, 3 studies [17–19] described masked endpoint assessment. One study [9] described blinded assessment of infections but did not describe blinded assessment of secondary outcomes, such as NIHSS and mRS. Blinding was not specified in one study [20]. Two studies [18, 20] did not report the losses of follow-up. One study [16] described the loss of follow-up, but did not mention the further details. Only per-protocol analysis was executed in one study [20]. The whole quality of all included studies was judged as moderate.

### Mortality

**Figure 3:**
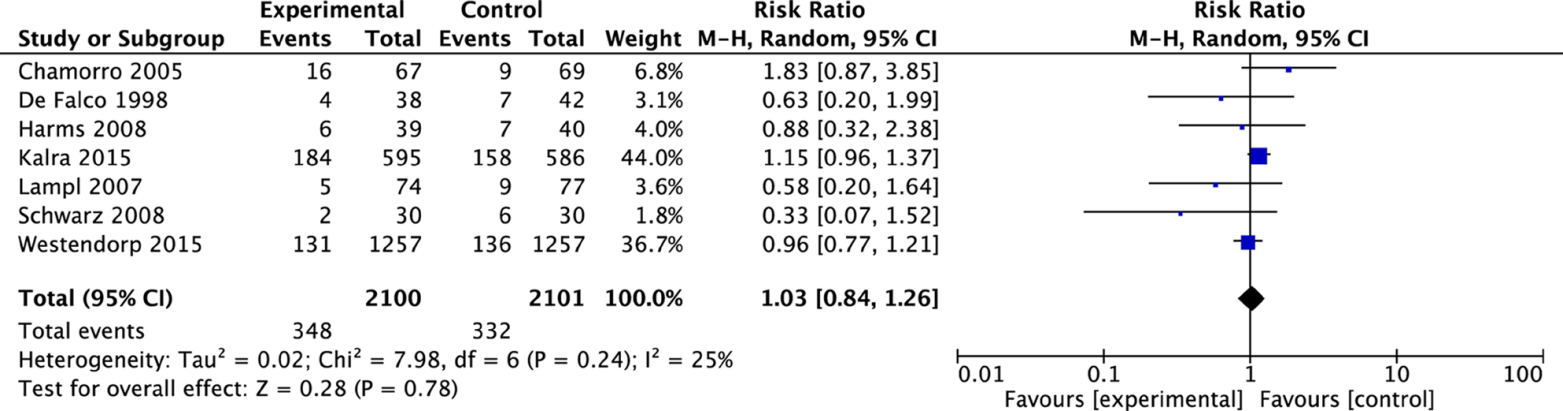
Meta-analysis on mortality The summary effect estimate (risk ratio, RR) for individual randomized controlled trials (RCTs) are indicated by green rectangles (the size of the rectangle is proportional to the study weight), with the black horizontal lines representing 95 per cent c.i. The overall summary effect estimate and 95 per cent c.i. are indicated by the black diamond below.

Results of all 7 studies were available for the analysis of mortality at the end of follow-up. Meta-analysis showed that antibiotic prophylaxis had no effect on overall mortality (RR = 1.03, 95% CI 0.84 to1.26, *p* = 0.78) (See Figure 3). There was no substantial heterogeneity for mortality (*I*^2^ = 25%, *p* = 0.24) (See Figure 3).

### Infection rate

**Figure 4:**
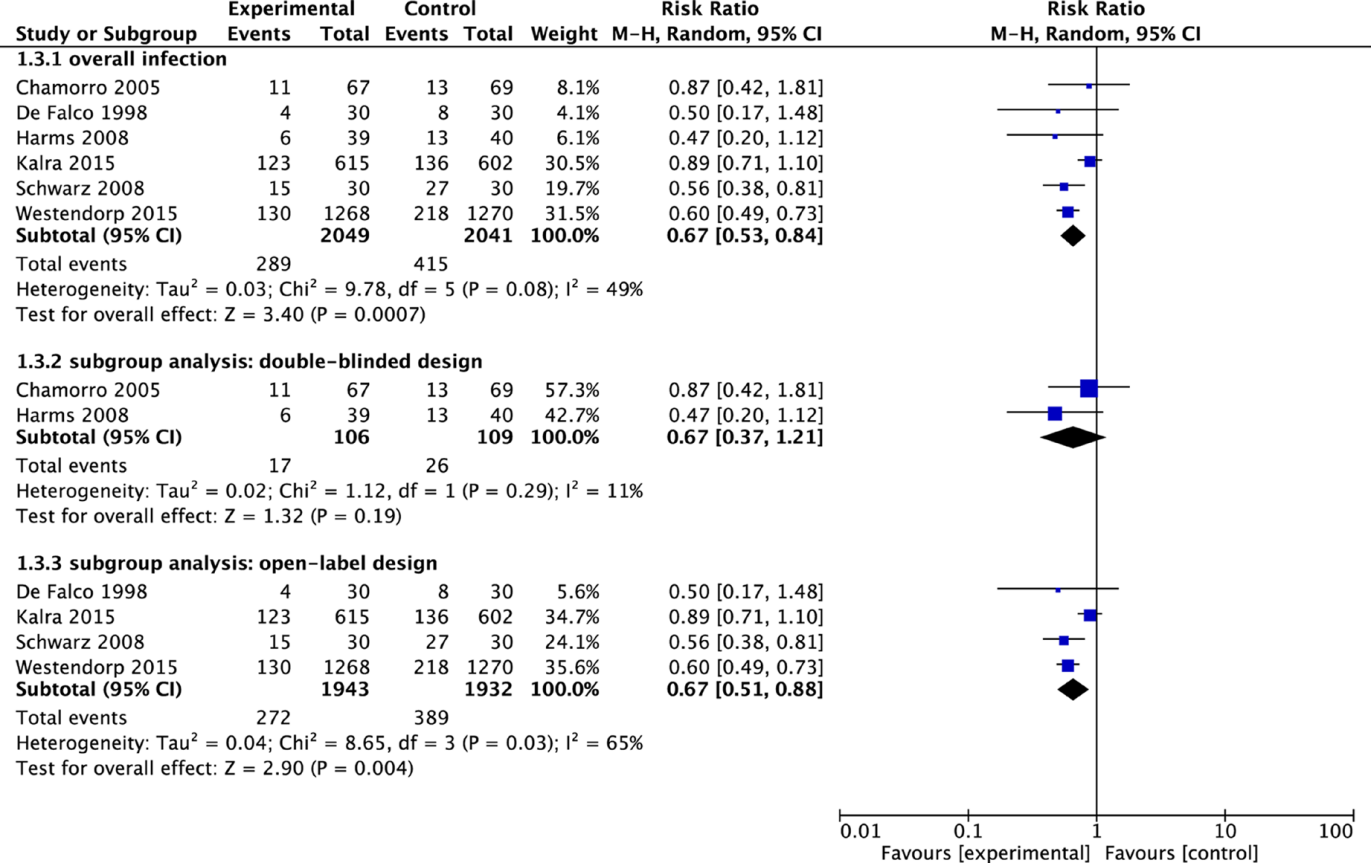
Meta-analysis on infection rate The summary effect estimate (risk ratio, RR) for individual randomized controlled trials (RCTs) are indicated by green rectangles (the size of the rectangle is proportional to the study weight), with the black horizontal lines representing 95 per cent c.i. The overall summary effect estimate and 95 per cent c.i. are indicated by the black diamond below.

The definitions for the diagnosis of infection differed substantially across trials (See Table 2). Thus, we used infection rate defined by the investigators. One study [18] did not report infection rate. The pooled effect size in the remaining 6 showed an association between antibiotic prophylaxis and reduced incidence of infection (RR = 0.67, 95% CI 0.53 to 0.84, *p* = 0.00) (See Figure 4). No significant heterogeneity was found among the identified comparisons (*I*^2^ = 49%, *P* = 0.08) (See Figure 4).

**Table 2:** Definitions used for infection

Source	Definition
Chamorro 2005^15^	Temperature > 37.5°C in two determinations; or > 37.8 in a single determination in patients with suggestive symptoms; white blood cell count > 11,000/mL or < 4000/mL; pulmonary infiltrate on chest x-rays, or cultures positive for a pathogen. Early infection: within 7 days, late: 8 to 90 days.
Harms 2008^16^	Pneumonia, > 1 of: abnormal respiratory examination, or pulmonary infiltrates in chest x-rays, productive cough with purulent sputum, microbiological cultures from lower respiratory tract or blood cultures, leukocytosis and elevation of CRP. UTI: > 1 of the following: fever (temperature > 38.0°C), urine sample positive for nitrite, leucocyturia, and significant bacteriuria.
Kalra 2015^17^	Criteria for pneumonia from the Centres for Disease Control and Prevention
De Falco 1998^20^	Infectious complications: bronchopulmonary, urinary or hyperthermia of unspecified origin. No definitions specified.
Lampl 2007^18^	Not evaluated.
Schwarz 2008^9^	Pneumonia: new infiltrate on chest x-ray compatible with the diagnosis of infection plus at least one of the following: fever (temperature > 38°C), leukocytosis > 12,000/μL or leukopenia < 3000/μL, purulent tracheal secretions Tracheobronchitis: purulent tracheals secretions or sputum plus at least 1 of the following: fever (temperature > 38°C), leukocytosis > 12,000/μL or leukopenia < 3000/μL UTI: > 25 leukocytes/μL in the urine if not explained by other findings. Bacteremia: bacteria in blood cultures Sepsis: clinical evidence of an infection with at least two of the following: temperatures > 38°C or < 35°C, tachycardia > 90/minute, tachypnoea > 20/minute, leukocytosis > 12,000/μL or leukopenia < 3000/μL Infection of unclear origin or other infections: clinical evidence of an infection of unknown origin or any other systemic infection
Westendorp 2015^19^	First, clinical diagnosis according to the treating physician will be recorded. Second, diagnosis of infection the modified criteria of the United States Centres for Disease Control and Prevention

Abbreviations: CRP, C-reactive protein; UTI, urinary tract infection.

### Poor functional outcome

**Figure 5:**
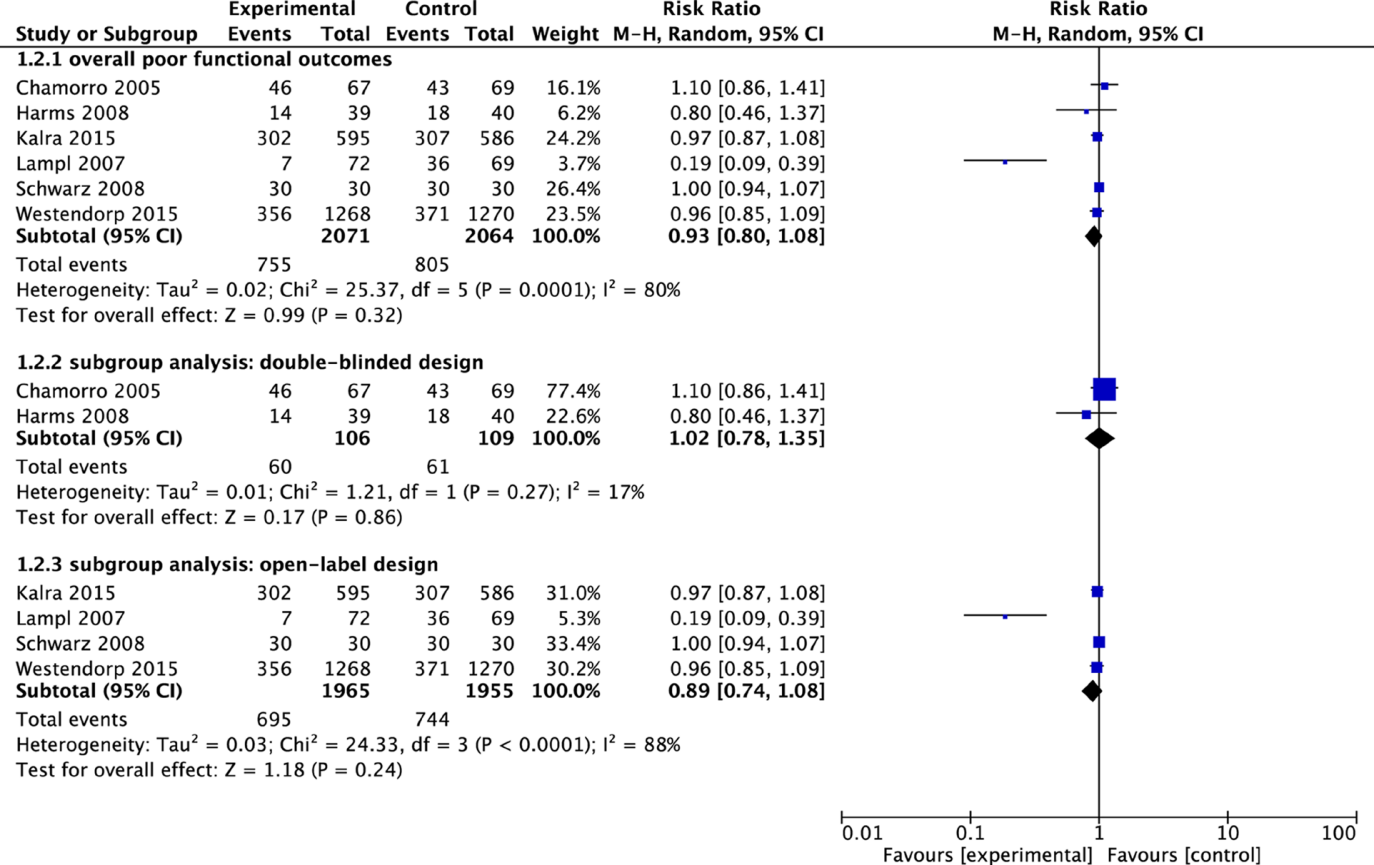
Meta-analysis on poor functional outcomes The summary effect estimate (risk ratio, RR) for individual randomized controlled trials (RCTs) are indicated by green rectangles (the size of the rectangle is proportional to the study weight), with the black horizontal lines representing 95 per cent c.i. The overall summary effect estimate and 95 per cent c.i. are indicated by the black diamond below.

One trial [20] did not report the number of patients with poor outcome at the endpoint assessment. So the data on poor functional outcome was available in 6 trials. The pooled RR of poor functional outcome was 0.93 (95% CI 0.80 to 1.08, *p* = 0.32) (See Figure 5). We identified the evidence of statistical heterogeneity (*I*^2^ = 80%, *p* < 0.00) (See Figure 5).

### Adverse events

Medication-related AEs were reported in 4 articles [9, 16, 17, 19]. In one study [16], AEs related to antibiotic were not described; however, one patient assigned in antibiotics prophylaxis group was reported to suffer from post-stroke pneumonia which was caused by methicillin-resistant *Staphylococcus aureus* (MRSA); however, the MRSA has been colonized before the patient enrolled into the present study. One study [9] reported that each participated patients experienced exanthema and elevated liver enzymes. The incidence of *Clostridium difficile* toxin (CDT)-positive diarrhea (2 of 615 versus 4 of 602) and MRSA colonization (11 of 615 versus 14 of 602) were low and equal between antibiotic prophylaxis and control groups respectively in one study [17]. One study [19] reported that seven and two patients who were assigned into antibiotics prophylaxis group experienced allergic reaction and *C. difficile* infection respectively.

### Subgroup analysis

We performed subgroup analysis according to the study design (double-blinded, open-label and unclear). Two trials [15, 16] used a double-blinded design, four trials [9, 17–19] used an open-label design and one trial [20] did not mention blind method. In the subgroup of using double-blinded design, there was no improvement in poor functional outcome (RR = 1.02, 95% CI 0.78 to 1.35, *p* = 0.86) with the insignificant statistical heterogeneity (*I*^2^ = 17%, *p* = 0.27) (See Figure 5). Infection rate was not significantly reduced in patients receiving antibiotic prophylaxis (RR = 0.67, 95% CI 0.37 to 1.21, *p* = 0.19) with the insignificant statistical heterogeneity (*I*^2^ = 11%, *p* = 0.29) (See Figure 4). In the subgroup of using open-label design, there was also no improvement in poor functional outcome (RR = 0.89, 95% CI 0.74 to 1.08, *p* = 0.24) with the significant statistical heterogeneity (*I*^2^ = 88%, *p* < 0.00) (See Figure 5). The pooled RR of infection rate showed there was association between the antibiotic prophylaxis and the reduced infection rate (RR = 0.67, 95% CI 0.51 to 0.88, *p* = 0.004) with the significant statistical heterogeneity (*I*^2^ = 65%, *p* = 0.03) (See Figure 4).

### Sensitivity analysis

**Figure 6:**
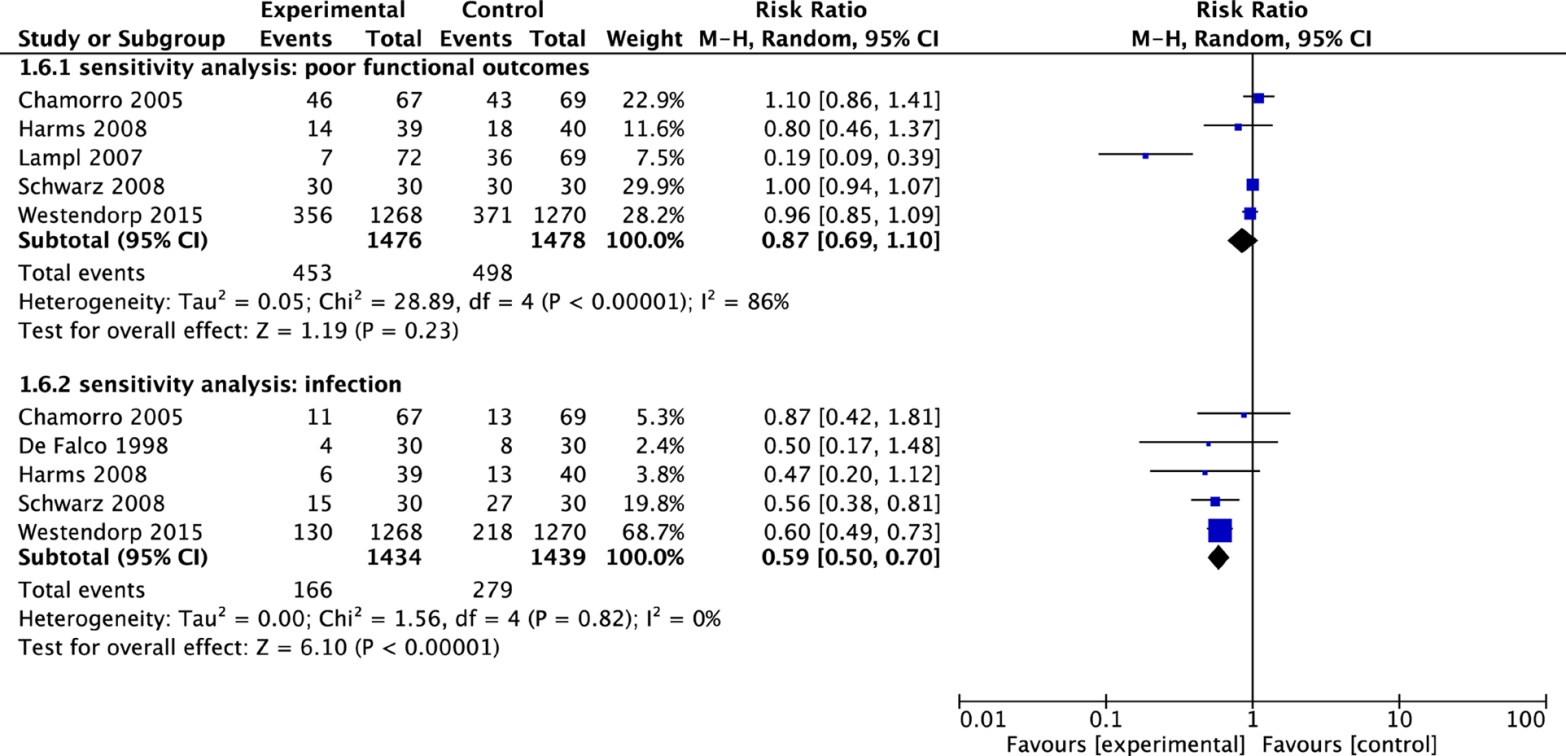
Sensitivity analysis The summary effect estimate (risk ratio, RR) for individual randomized controlled trials (RCTs) are indicated by green rectangles (the size of the rectangle is proportional to the study weight), with the black horizontal lines representing 95 per cent c.i. The overall summary effect estimate and 95 per cent c.i. are indicated by the black diamond below.

One study [17] used cluster-randomized design which could introduce dependence (or clustering) between individual units sampled. The exclusion of this study did not change the pooled RR of poor functional outcome (RR = 0.87, 95% CI 0.69–1.10, *p* = 0.27) nor did it reduce heterogeneity (*I*^2^ = 86%) (See Figure 6) substantially. Moreover, the sensitivity analysis was conducted by excluding the same study and a robust pooled result in infections was showed (RR = 0.59, 95% CI 0.50–0.70, *p* < 0.00001) (See Figure 6).

## DISCUSSION

Our present meta-analysis demonstrated that the administration of antibiotic prophylaxis did reduce post-stroke infection rate from 14% to 20%. Prophylactic antibiotic did not affect the mortality and poor functional outcome in patients with acute stroke. Sensitivity analyses showed that infection rate and poor functional outcome were stable. We have found no major AEs of prophylactic antibiotic treatment.

A previous Cochrane review enrolled 5 RCTs showed that antibiotic prophylaxis reduced the infection rate without major AEs [8]. However, the conclusion of this review generated inconclusive findings due to insufficient accumulated sample size of 506 patients [8]. The conclusion was also outdated because of more new RCTs have been carried out since then. Our study included two new large-scale trials and reevaluated the efficacy and safety of prophylactic antibiotic in acute stroke patients.

Infections are frequent after stroke. Despite general measures to prevent infections after stroke onset, pneumonia and UTI remains a common and severe clinical problem even for patients treated in stroke units [7, 22]. Pneumonia can be classified into early (within the first week) and late pneumonia (after the first week) after stroke [23]. Early pneumonia is endogenous and is caused by normal flora carried in the oropharynx on hospital admission. While, delayed pneumonia is resulted from abnormal flora which was acquired in the oropharynx during hospitalization. Recently, a new consensus was reached on the diagnosis of stroke-associated pneumonia: lower respiratory tract infections within the first 7 days after stroke onset [24]. Patients with stroke are vulnerable to UTI due to increased risk of bladder dysfunction, immunosuppression and increased Foley catheter use [23, 25]. Infections other than pneumonia and UTI occurred in 13% of stroke patients and included such as skin and intravenous line infections, sepsis and cholecystitis [26]. Many reasons for infection have been postulated, including older age, male sex, stroke severity, dysphagia, gastrointestinal dysmotility and stroke-induced immunodepressive state [6, 27, 28], and so on. Stroke-induced immunodeficiency via the hypothalamic axis and the sympathetic nervous system can be detected within a few hours after cerebral ischemia and may persist over a few weeks [29]. The immunosuppression may increase the susceptibility to the infections in patients after stroke [29]. Fever and systemic inflammatory response associated with infections may influence stroke recovery [30]. Infections, especially pneumonia usually lead to increased mortality and poor functional outcome [6, 25, 26]. Therefore, there is strong rationale to investigate the effect of antibiotic prophylaxis on patients with acute stroke.

The evidence included in the present meta-analysis showed that preventive antibiotic therapy reduced the occurrence of infection. Several issues could confound the results. Firstly, only one study by Kalra [17] did not show reduced post-stroke pneumonia. One possible reason could be the high-quality care provided at the stroke units participating in the trial. Post-stroke pneumonia is mostly due to aspiration of oropharyngeal secretions promoted by dysphagia, dependent feeding, teeth decay and reduced or ineffective cough. Preventive antibiotic therapy did not add to existing preventive measures including regular suction, positioning, modified diets and early search of infections to start antibiotic treatment [31]. Secondly, definition of infection differed substantially among included studies. One study [20] did not describe the definition of infection in detail. In three studies [16, 17, 19], diagnosis of infection was judged by using criteria of the United States Centers for Disease Control and Prevention [4]. Less strict definitions could overestimate the number of infections, especially in open label studies. Thirdly, to prevent infections, the antibiotic should cover the common causative organisms in post-stroke infections such as pneumonia and urinary tract infections. The most common causative bacteria of pneumonia are *taphylococcus aureus* and gram-negative bacteria such as *Klebsiella pneumoniae, Pseudomonas aeruginosa, Escherichia coli* or *Enterobacter spp* [5]. *Escherichia coli* and *Staphylococcus saprophyticus* are commonly identified in patients with urinary tract infection [5]. Antibiotics in six trials [9, 15–17, 19, 20] covered these most common bacteria. However, minocycline in one study [18] inadequately covered the antimicrobial spectrum in patients with acute stroke. The main aim of this study was to investigate a possible neuroprotective effect of minocycline, not to assess the role of this medicine in prevention of the post-stroke infection. Another potential neuroprotective antibiotic used in one study [19] is ceftriaxone, which has a combination of effective coverage of antimicrobial spectrum and neuroprotective properties [19]. Yet, it still failed at the confirmatory trial stage.

The result of this meta-analysis indicated that preventive antibiotic had no effect on overall post-stroke mortality. Several factors might influence the results. As we know, mortality in acute stroke varied between 15% and 25% [32]. However, most of included trials [9, 15, 16, 18–20] had low mortality (0%–15%) and only one trial [17] had relatively high mortality (29%). Three studies exclude patients with a short life expectancy. Inclusion of less severe patients in this meta-analysis might overestimate the efficacy of antibiotic prophylaxis due to the low mortality. Severe patients might benefit the most from preventive antibiotic treatment. Infection rate was associated with the patients’ clinical condition. Studies including patients with a higher stroke severity or lower levels of consciousness showed higher infection rates, in particular for pneumonia [5]. Another possibility is that antibiotic can prevent the early or mild infections from happening, but for those patients with severe infections, it might also be too severe for antibiotic prophylaxis to reduce the mortality.

Several studies showed that infections were associated with mortality and poor functional outcome [6, 25, 26]. Antibiotic did reduce the post-stroke infection in our meta-analysis, but gave no reason for the fact that decrease of infection rate did not result into a decreased mortality or improve in functional outcome. Poor functional outcome (dependency) was measured with mRS or BI in activities of daily living. Poor functional outcome is the most important measure of outcome since the aim of therapy should not only be to reduce death but also to reduce disability and dependency in survivors. Infections could affect outcome in several ways. Firstly, they lead to immobilization, general frailty and a delay in rehabilitation due to prolonged hospital stay [5, 33]. More importantly, immunological effects of infections could worsen outcome. Evidence from experimental studies suggests that infection promotes antigen presentation and autoimmunity against the brain [34]. Post-stroke infections include pneumonia, UTI, skin and intravenous line infections, sepsis, cholecystitis, etc. A recent study implied that UTI was not associated with functional outcome [26]. Pneumonia is a well-recognized predictor of poor outcome in stroke patients [6, 7, 25, 26]. Functional outcome after a stroke is determined largely by the nature of incident stroke, age and premorbid function [26]. Post-stroke pneumonia might be a marker or bystander of stroke severity, and prophylactic antibiotic might not change the course of disease [19]. Infections after stroke most likely result from complex interactions of bacterial, chemical, mechanical (e.g., indwelling catheters), and immunological mechanisms that might not be prevented by antibiotics alone [17] It is therefore possible that toxic effects of treatment as well as the infections themselves mediate the poor outcomes seen in infected stroke patients [35]. To our knowledge, a comprehensive analysis of outcome and infectious complications in stroke patients has not become available in the literature. Moreover, in real-world practice, when diagnosed post-stroke infection in control group patients, early treatment might be just as effective as preventive antibiotic treatment.

Like all medicines, antibiotics may have the potential to cause adverse effects including gastrointestinal problems, allergic reactions, bone marrow suppression, ototoxicity, neurotoxicity and nephrotoxicity. Antibiotic resistance can be another unintended effect of taking antibiotics. Our results suggested that few adverse events occurred in both treatment groups and the incidence of adverse events was low and equal in both groups basically. At least, preventive antibiotic treatment is safe.

Two trials [15, 16] used a double-blinded design and four trials [9, 17–19] used an open-label design. The open-label trial is a type of clinical trial in which both the researchers and participants know which treatment is being administered. The open-intervention allocation can influence physician diagnosis of post-stroke infections and other outcomes, which could lead to an overestimation of a possible effect. This detection bias might have been minimized by use of blinded assessment of endpoint. Hence, the sub-analysis of trial design indicated that the pooled results were robust.

We have to consider the possibility that antibiotics of different classes may differentially affect stroke outcome independent of infection. For example, both β-lactam antibiotics and minocycline have neuroprotective properties [36], while fluoroquinolone antibiotics may be neurotoxic. Zierath [37] designed a study which compared behavioral and histological outcomes from stoke in Lewis rats treated with ceftiofur and enrofloxacin. They found no individual antibiotic class was associated with a functional benefit, but rats which were given enrofloxacin had significantly worse functional outcomes. A clinical trial [15] produced the similar result that levofloxacin did not prevent infection and was associated with worse outcome. The neurotoxic mechanisms of Fluoroquinolone is unclear and maybe referred to the inhibition of γ-aminobutyric acid- A receptor binding, and action at the *N*-methyl-D-aspartate receptor or alteration of K+ currents [37]. Several studies have showed neuroprotective effects of minocycline and ceftriaxone, yet neither has succeeded at the confirmatory trial stages [19, 38]. Although no benefit of antibiotics was proved in the treatment of patients with acute stroke, the potential neuroprotective properties confirmed in preclinical studies deserve more investigation. Given the existing evidence that antibiotic administration is safe and well tolerated, the neuroprotective potential of antibiotics should be explored continually in future studies. Furthermore, since the immunological mechanisms and immunodepression take effect immediately and last for a few weeks after the stroke, the time window before start of preventive antibiotic therapy (up to 48 hours) might be too long, and the duration of preventive antibiotic therapy might be too short [39].

Several potential limitations should be appreciated in our analysis. First of all, the conclusion provided in a meta-analysis is only as reliable as the methods used to assess the effect in the primary studies [40]. It also does not overcome problems which inherent in the design of eligible studies. The heterogeneity should be considered, which included the difference in the types and severity of stroke patients, the dose, route and duration of different antibiotics and the diagnostic criteria used for infection. For future studies, standardized definitions of post-stroke infection and time of follow-up are preferable. Secondly, the included studies used insensitive measure methods. Current researchers employed BI and mRS as the primary measure of functional recovery. However, sensitivity of BI and mRS [41, 42] is poor across the range of possible outcomes, particularly in minor or more severe strokes. So multidimensional approaches used to evaluate stroke recovery and reliable biomarkers used to objectively evaluate the efficacy of interventions are necessary for future large clinical trials. Thirdly, despite our extensively searching for relevant studies using multiple databases and multiple search items; however, there were difficulties in obtaining unpublished studies. Considering the fact that unpublished trials were mostly those with negative results, unpublished data might just strengthen rather than alter the negative results of this meta-analysis [43]. Fourth, we restricted our search to studies published in English, which potentially led to language bias. Fifth, the findings of present meta-analysis might be impacted by stroke type, stroke severity and patient's age. Unfortunately, we did not perform subgroup analysis according to stroke type, stroke severity and patient's age due to lack of detailed data. Given that only 418 patients with hemorrhagic stroke were included in three studies [15, 17, 19], it may be necessary to further investigate the efficacy of antibiotics prophylaxis in the treatment of acute hemorrhagic stroke. Fifth, publication bias and meta-regression for included studies was not detected because of the limited number (below < 10) of eligible studies, which might influence the results. Sixth, theoretically, studies with a more tailored approach using stricter inclusion criteria (e.g., the early use of antibiotics with adequate duration, more severe patients) and including biomarkers might identify patients who could benefit from preventive antibiotic therapy after stroke. Thus, larger RCTs are required to initiate in the near future. Seventh, “infection” and “acute stroke” had different and heterogeneous definitions in the included different studies and thus readers should to interpret the meta-analysis results with caution.

## MATERIALS AND METHODS

We designed and performed this systematic review and meta-analysis and reported the results according to Cochrane Handbook for systematic review and the Preferred Reporting Items for Systematic Reviews and Meta-analysis (PRISMA) statement [44] respectively. The protocol was prospectively registered at PROSPERO online database and a registration number of CRD42015026980 has been assigned (available at: http://www.crd.york.ac.uk/prospero/). The informed consent and ethical approval were not required because of this present study was conducted based upon the information from previous studies.

### Eligibility criteria

We designed inclusion criteria based on PICOS principle as following: (1) study design: RCTs investigating the efficacy of antibiotic prophylaxis versus comparisons including placebo or standard treatment; (2) eligible participants: acute stroke patients aged 18 years and older; (3) intervention: antibiotic prophylaxis was administered within 48 hours after stroke onset; and (4) outcomes: reported at least one of outcomes of interest including infection or mortality.

Studies met following criteria were excluded from the present study: studies published in non-English language; the sample size of less than 20 patients in each group; non-original researches such as reviews, expert opinions, letters, commentaries, editorials and research protocol.

### Search methods

We designed two-step search strategy for capturing all potential citations. (1) Three target databases including PubMed, EMBASE and the Cochrane library were electronically searched, and then the reference lists of eligible articles were checked after screened all records identified at initial search period. The initial search was performed on August 2015 and the updated notification was custom daily until September 2015. Following terms were used: ‘stroke’, ‘brain ischemia’, ‘cerebral ischemia*’, ‘brain hemorrhage*’, ‘cerebral stroke*’, ‘cerebral infarction’, ‘intracranial hemorrhages’, ‘cerebrovascular accident’, ‘antibiotic prophylaxis’ and ‘preventive antibiotic’. We used Boolean operators (OR, AND, and NOT) constructed all search algorithms. Languages restriction was not imposed in our study. We documented all search algorithms in electronic supplementary material (Supplementary Material of Search Algorithm).

### Study selection

Two independent authors firstly judged the eligibility of the relevant articles through screening titles and abstracts, and checking full-text to determine whether a certain study can be included. Any disagreements on eligibility were resolved by consulting a third reviewer until a consensus was reached.

### Data extraction

Two investigators independently abstracted data from all eligible studies using a predesigned standard data extraction form. Following information including first author's name, year of publication, inclusion and exclusion criteria sample size, intervention and outcomes of interest were abstracted. Discrepancies were resolved in consultation with a third reviewer.

### Assessment of risk of bias

The Cochrane Risk of Bias tool was used to appraise the risk of bias of all included studies [45]. The following domains were evaluated accordingly: random sequence generation, allocation concealment, blinding (participants, personnel and outcome assessment), incomplete outcome data, selective reporting and other sources of bias [45]. Based upon the matching level between abstracted information and judgment criteria, each study might be rated to be as low, unclear or high risk of bias [45]. A third author will be consulted if any divergence was identified.

### Outcomes measures

#### Primary outcomes

(1) mortality at the end of follow-up and (2) infection rate within the first 2 weeks after stroke onset.

#### Secondary outcomes

(1) poor functional outcome (dependency): defined as a Barthel Index (BI) < 60 or a score on modified Rankin score (mRS) > 2 at the end of follow-up; and (2) medication-related adverse events (AEs).

### Statistical analysis

We used Review Manager (RevMan) version 5.3.5 (Cochrane Collaboration, Copenhagen, Denmark) to perform all meta-analyses. We estimated the risk ratio (RR) with 95% confidence interval (CI) to express dichotomous outcomes including mortality, infection and poor functional outcome. The DerSimonian-Laird random-effect model accounting for different sources of variation from studies was used as a conservative approach to calculate effect estimates. Heterogeneity across studies was examined with inconsistency analysis (*I*^2^) and *Q*-test (with a *P*-value < 0.10 considered substantial) [46]. A *I*^2^ < 50% shows homogeneous, a *I*^2^ ≥ 50% indicates the existence of heterogeneity. We performed subgroup analyses according to study design (double-blinded and open-label). Moreover, we carried out sensitivity analyses based on study design. All reported *P* values were 2-tailed and a 0.05 was seen to be significance. The publication bias and meta-regression will not be evaluated when the number of eligible studies was less than 10.

## CONCLUSIONS

In adults with acute stroke, antibiotic prophylaxis did reduce the occurrence of infection, but did not reduce mortality and poor functional outcome. Sensitivity analysis showed that infection rate and poor functional outcome were stable. Thus, antibiotic prophylaxis may not be recommended for acute stroke patients. No major side-effects of preventive antibiotic therapy were reported.

## SUPPLEMENTARY MATERIALS



## Abbreviations

RCTs: randomized controlled trials
PRISMA: Preferred Reporting Items for Systematic Reviews and Meta-analysis
MeSH: medical subject heading
BI: Barthel Index
mRS: modified Rankin score
AEs: adverse events
RevMan: Review Manager
RR: risk ratio
CI: confidence interval
I^2^: inconsistency analysis
NIHSS: National Institute of Health Stroke Scale
CNS: Canadian Neurological Scale
ID: identity card
MRSA: methicillin-resistant *Staphylococcus aureus*
CDT: *Clostridium difficile* toxin
UTI: urinary tract infection
