# Supplementary material for: Antibiotic prophylaxis for infections in patients with acute stroke: a systematic review and meta-analysis of randomized controlled trials
Source: Oncotarget. 2017 Jul 6;8(46):81075–87. doi: 10.18632/oncotarget.19039 (PMC5655263; doi:10.18632/oncotarget.19039)
Supplement: Supplementary file 1 [file oncotarget-08-81075-s001.pdf]

Antibiotic prophylaxis for infections in patients with acute stroke: a systematic review and meta-analysis of randomized controlled trials

Supplementary Materials

PubMed

((((((((((randomized controlled trial[Publication Type] OR controlled clinical trial[Publication Type]) OR randomized[Title/Abstract]) OR placebo[Title/Abstract]) OR "drug therapy"[Subheading]) OR random*[Title/Abstract]) OR trial[Title/Abstract]) OR groups[Title/Abstract]) NOT ("animals"[MeSH Terms] NOT "humans"[MeSH Terms])))) AND (((((((((premedicat*[Title/Abstract]) OR prophyla*[Title/Abstract]) OR prevent*[Title/Abstract])) AND (((((bacteriocid*[Title/Abstract]) OR antibiotic*[Title/Abstract]) OR anti bacterial[Title/Abstract]) OR antibacterial[Title/Abstract]) OR "Anti-Bacterial Agents"[Mesh]))) OR "Antibiotic Prophylaxis"[Mesh])) AND ((((((((((((brain hemorrhage*[Title/Abstract]) OR cerebral hemorrhage*[Title/Abstract]) OR cerebral ischemia*[Title/Abstract]) OR cerebral stroke*[Title/Abstract]) OR cerebrovascular accident*[Title/Abstract]) OR cerebral infarction[Title/Abstract]) OR brain infarction[Title/Abstract]) OR Intracranial Hemorrhage*[Title/Abstract]) OR Brain Ischemia[Title/Abstract]) OR Stroke[Title/Abstract]) OR "Brain Ischemia"[Mesh]) OR "Stroke"[Mesh])).

Cochrane library

#1 MeSH descriptor: [Stroke] explode all trees
#2 MeSH descriptor: [Brain Ischemia] explode all trees
#3 MeSH descriptor: [Intracranial Hemorrhages] explode all trees
#4 Stroke:ti,ab,kw (Word variations have been searched)
#5 #1 or #2 or #3 or #4
#6 MeSH descriptor: [Antibiotic Prophylaxis] explode all trees
#7 prevent* antibiotic*:ti,ab,kw (Word variations have been searched)
#8 #6 or #7
#9 #5 and #8

EMBASE

#51. 'basal ganglion hemorrhage'/exp OR 'brain hematoma'/exp OR 'brain hemorrhage'/exp OR 'brain infarction'/exp OR 'cerebrovascular accident'/exp OR stroke:ab,ti OR 'brain hemorrhage':ab,ti OR 'basal ganglion hemorrhage':ab,ti OR 'brain hematoma':ab,ti OR 'brain infarction':ab,ti OR 'cerebrovascular accident':ab,ti OR 'brain ischemia'/exp OR 'brain ischemia':ab,ti OR (intracranial AND hemorrhage*:ab,ti) OR (cerebral AND infarction:ab,ti) OR (cerebrovascular AND accident*:ab,ti) OR (cerebral AND stroke*:ab,ti) OR (cerebral AND ischemia*:ab,ti) OR (cerebral AND hemorrhage*:ab,ti) OR (brain AND hemorrhage*:ab,ti) AND ('antibiotic prophylaxis'/exp OR ('antibiotic agent'/exp OR antibacterial:ab,ti OR (anti AND bacterial:ab,ti) OR antibiotic*:ab,ti OR bacteriocid*:ab,ti AND ('prophylaxis'/exp OR prevent*:ab,ti OR prophyla*:ab,ti OR premedicat*:ab,ti))) AND ('randomized controlled trial'/exp OR 'controlled clinical trial'/exp OR 'randomized controlled trial':ab,ti OR random*:ab,ti) NOT ('animal'/exp NOT ('animal'/exp AND 'human'/exp)) AND ([embase]/lim OR [embase classic]/lim)

#50. 'basal ganglion hemorrhage'/exp OR 'brain hematoma'/exp OR 'brain hemorrhage'/exp OR 'brain infarction'/exp OR 'cerebrovascular accident'/exp OR stroke:ab,ti OR 'brain hemorrhage':ab,ti OR 'basal ganglion hemorrhage':ab,ti OR 'brain hematoma':ab,ti OR 'brain infarction':ab,ti OR 'cerebrovascular accident':ab,ti OR 'brain ischemia'/exp OR 'brain ischemia':ab,ti OR (intracranial AND hemorrhage*:ab,ti) OR (cerebral AND infarction:ab,ti) OR (cerebrovascular AND accident*:ab,ti) OR (cerebral AND stroke*:ab,ti) OR (cerebral AND ischemia*:ab,ti) OR (cerebral AND hemorrhage*:ab,ti) OR (brain AND hemorrhage*:ab,ti) AND ('antibiotic prophylaxis'/exp OR ('antibiotic agent'/exp OR antibacterial:ab,ti OR (anti AND bacterial:ab,ti) OR antibiotic*:ab,ti OR bacteriocid*:ab,ti AND ('prophylaxis'/exp OR prevent*:ab,ti OR prophyla*:ab,ti OR premedicat*:ab,ti))) AND ('randomized controlled trial'/exp OR 'controlled clinical trial'/exp OR 'randomized controlled trial':ab,ti OR random*:ab,ti) NOT ('animal'/exp NOT ('animal'/exp AND 'human'/exp))

#49. 'randomized controlled trial'/exp OR 'controlled clinical trial'/exp OR 'randomized controlled trial':ab,ti OR random*:ab,ti NOT ('animal'/exp NOT ('animal'/exp AND 'human'/exp))

#48. 'animal'/exp NOT ('animal'/exp AND 'human'/exp)
#47. 'animal'/exp AND 'human'/exp
#46. 'human'/exp
#45. 'animal'/exp
#44. 'randomized controlled trial'/exp OR

‘controlled clinical trial’/exp OR ‘randomized controlled trial’:ab,ti OR random*:ab,ti

#43. random*:ab,ti

#42. ‘randomized controlled trial’:ab,ti

#40. ‘controlled clinical trial’/exp

#39. ‘randomized controlled trial’/exp

#38. ‘basal ganglion hemorrhage’/exp OR ‘brain hematoma’/exp OR ‘brain hemorrhage’/exp OR ‘brain infarction’/exp OR ‘cerebrovascular accident’/exp OR stroke:ab,ti OR ‘brain hemorrhage’:ab,ti OR ‘basal ganglion hemorrhage’:ab,ti OR ‘brain hematoma’:ab,ti OR ‘brain infarction’:ab,ti OR ‘cerebrovascular accident’:ab,ti OR ‘brain ischemia’/exp OR ‘brain ischemia’:ab,ti OR (intracranial AND hemorrhage*:ab,ti) OR (cerebral AND infarction:ab,ti) OR (cerebrovascular AND accident*:ab,ti) OR (cerebral AND stroke*:ab,ti) OR (cerebral AND ischemia*:ab,ti) OR (cerebral AND hemorrhage*:ab,ti) OR (brain AND hemorrhage*:ab,ti) AND (‘antibiotic prophylaxis’/exp OR (‘antibiotic agent’/exp OR antibacterial:ab,ti OR (anti AND bacterial:ab,ti) OR antibiotic*:ab,ti OR bacteriocid*:ab,ti AND (‘prophylaxis’/exp OR prevent*:ab,ti OR prophyla*:ab,ti OR premedicat*:ab,ti)))

#37. ‘antibiotic prophylaxis’/exp OR (‘antibiotic agent’/exp OR antibacterial:ab,ti OR (anti AND bacterial:ab,ti) OR antibiotic*:ab,ti OR bacteriocid*:ab,ti AND (‘prophylaxis’/exp OR prevent*:ab,ti OR prophyla*:ab,ti OR premedicat*:ab,ti))

#36. ‘antibiotic agent’/exp OR antibacterial:ab,ti OR (anti AND bacterial:ab,ti) OR antibiotic*:ab,ti OR bacteriocid*:ab,ti AND (‘prophylaxis’/exp OR prevent*:ab,ti OR prophyla*:ab,ti OR premedicat*:ab,ti)

#35. ‘prophylaxis’/exp OR prevent*:ab,ti OR prophyla*:ab,ti OR premedicat*:ab,ti

#34. premedicat*:ab,ti

#33. prophyla*:ab,ti

#32. prevent*:ab,ti

#31. ‘prophylaxis’/exp

#30. ‘antibiotic agent’/exp OR antibacterial:ab,ti OR (anti AND bacterial:ab,ti) OR antibiotic*:ab,ti OR bacteriocid*:ab,ti

#29. bacteriocid*:ab,ti

#28. antibiotic*:ab,ti

#27. anti AND bacterial:ab,ti

#26. antibacterial:ab,ti

#25. ‘antibiotic agent’/exp

#24. ‘antibiotic prophylaxis’/exp

#23. ‘basal ganglion hemorrhage’/exp OR ‘brain hematoma’/exp OR ‘brain hemorrhage’/exp OR ‘brain infarction’/exp OR ‘cerebrovascular accident’/exp OR stroke:ab,ti OR ‘brain hemorrhage’:ab,ti OR ‘basal ganglion hemorrhage’:ab,ti OR ‘brain hematoma’:ab,ti OR ‘brain infarction’:ab,ti OR ‘cerebrovascular accident’:ab,ti OR ‘brain ischemia’/exp OR ‘brain ischemia’:ab,ti OR (intracranial AND hemorrhage*:ab,ti) OR (cerebral AND infarction:ab,ti) OR (cerebrovascular AND accident*:ab,ti) OR (cerebral AND stroke*:ab,ti) OR (cerebral AND ischemia*:ab,ti) OR (cerebral AND hemorrhage*:ab,ti) OR (brain AND hemorrhage*:ab,ti)

#22. brain AND hemorrhage*:ab,ti

#21. cerebral AND hemorrhage*:ab,ti

#20. cerebral AND ischemia*:ab,ti

#19. cerebral AND stroke*:ab,ti

#18. cerebrovascular AND accident*:ab,ti

#17. cerebral AND infarction:ab,ti

#16. intracranial AND hemorrhage*:ab,ti

#15. ‘brain ischemia’:ab,ti

#14. ‘brain ischemia’/exp

#13. ‘cerebrovascular accident’:ab,ti

#12. ‘brain infarction’:ab,ti

#11. ‘brain hematoma’:ab,ti

#10. ‘basal ganglion hemorrhage’:ab,ti

#9. ‘brain hemorrhage’:ab,ti

#6. stroke:ab,ti

#5. ‘cerebrovascular accident’/exp

#4. ‘brain infarction’/exp

#3. ‘brain hemorrhage’/exp

#2. ‘brain hematoma’/exp

#1. ‘basal ganglion hemorrhage’/exp
